# Node Self-Deployment Algorithm Based on an Uneven Cluster with Radius Adjusting for Underwater Sensor Networks

**DOI:** 10.3390/s16010098

**Published:** 2016-01-14

**Authors:** Peng Jiang, Yiming Xu, Feng Wu

**Affiliations:** 1 College of Automation, Hangzhou Dianzi University, Hangzhou 310018, China; xymhdu@163.com (Y.X.); fengwu@hdu.edu.cn (F.W.); 2 Key Lab for IOT and Information Fusion Technology of Zhejiang, Hangzhou 310018, China

**Keywords:** node self-deployment, uneven clustering, radius adjusting, network reliability

## Abstract

Existing move-restricted node self-deployment algorithms are based on a fixed node communication radius, evaluate the performance based on network coverage or the connectivity rate and do not consider the number of nodes near the sink node and the energy consumption distribution of the network topology, thereby degrading network reliability and the energy consumption balance. Therefore, we propose a distributed underwater node self-deployment algorithm. First, each node begins the uneven clustering based on the distance on the water surface. Each cluster head node selects its next-hop node to synchronously construct a connected path to the sink node. Second, the cluster head node adjusts its depth while maintaining the layout formed by the uneven clustering and then adjusts the positions of in-cluster nodes. The algorithm originally considers the network reliability and energy consumption balance during node deployment and considers the coverage redundancy rate of all positions that a node may reach during the node position adjustment. Simulation results show, compared to the connected dominating set (CDS) based depth computation algorithm, that the proposed algorithm can increase the number of the nodes near the sink node and improve network reliability while guaranteeing the network connectivity rate. Moreover, it can balance energy consumption during network operation, further improve network coverage rate and reduce energy consumption.

## 1. Introduction and Related Works

Underwater wireless sensor networks (UWSNs) are network-monitoring systems consisting of sensor nodes with the capabilities of perception, acoustic communication and computing in an underwater environment in a self-organized manner; their function is to transmit sensed information to a sink node for processing and analysis [1,2]. UWSNs can be applied to water environment monitoring, underwater exploration, marine military defense and other fields [3,4,5]. This type of network has become one of the most significant topics in the information field. Research on UWSNs mainly involves node deployment, node localization, time synchronization, network protocol design, *etc.* [6,7,8,9]. Among these topics, node deployment, as the foundation work for UWSNs design, has a direct effect on the quality of target coverage and network service.

The node-deployment problem refers to moving sensor nodes to their positions in an artificial or a self-organized manner to form a network topology that has special characteristics and can benefit future works [10]. According to the mobility capability of nodes, node deployment can be divided into static deployment, free-to-move node self-deployment and move-restricted node self-deployment [11,12,13,14,15,16]. Static deployment assumes that nodes do not have the capability to move and need to use artificial methods to deploy [17]. It is usually a centralized algorithm, needing the *a priori* environment information to calculate the pre-deployment positions; thus, it is inappropriate in many UWSN cases, such as underwater resource exploration and marine military defense [4]. Free-to-move node self-deployment assumes that nodes have the capability to move freely and can move in all directions [2]. Move-restricted node self-deployment assumes that nodes have the capability to move only vertically and can adjust their depth by themselves [18]. These two algorithms do not need any *a priori* information, so they are applied more widely. However, the former, needing some autonomous underwater vehicles to assist during the deployment process, leads to high energy and resource costs, as well as high operation difficulty [16,19,20]. On the contrary, the latter, having developed a simple underwater sensor node with vertical mobility by the method of filling in or drawing off water, operates simply and availably [21,22]. Thus, move-restricted node self-deployment is more practical than free-to-move node deployment.

On the one hand, a number of related studies have been conducted on the method of move-restricted node self-deployment. Wu *et al.* [23] proposed a Voronoi-based depth-adjustment scheme for UWSNs. To maximize the network coverage rate, this scheme compares the Voronoi region area of every node in the same layer to determine the nodes that should go down to the next layer. This process continues layer by layer until the nodes in the last layer have been determined. However, this method uses a centralized manner to adjust node depth, which is difficult to achieve in practice. Akkaya *et al.* [24] proposed a distributed self-deployment depth-adjustment algorithm to adjust the depth of nodes after their initial deployment to reduce the coverage overlaps between two neighboring nodes. Nodes continue to adjust their depth until the sensor coverage can no longer be improved. Du *et al.* [25] proposed a coverage algorithm based on fixed-directional movement for underwater sensor networks; this algorithm uses virtual forces to adjust a node to a position with zero joint force exertion and achieves node intelligent deployment in an independent manner. However, the aforementioned algorithms all view the network coverage rate as a standard and ignore the effect of the network connectivity rate on the network quality of service. Senel *et al.* [26] proposed the connected dominating set (CDS) based depth computation algorithm (CDA) on the basis of [24]. The algorithm initially constructs a connected backbone and then uses the dominator node on the backbone to individually optimize the position of its non-dominators iteratively. The algorithm ensures full-network connectivity while maximizing network coverage and reducing network deployment consumption. However, similar to other algorithms, CDA considers fixed communication radius nodes as deployment objects, which makes the nodes not be able to select an appropriate position flexibly during the process of node depth adjustment. CDA also views the network coverage rate or network connectivity rate as the criterion to deploy nodes and does not consider the number of nodes deployed within a certain range around the sink node. Those two increase network energy consumption and decrease network performance. In addition, the algorithm (*i.e.*, CDA) considering both the network coverage rate and the network connectivity rate, only considers the redundant coverage rate of the location where the distance between the node and its next-hop node is *R_c_* during the process of optimizing the node position; thus, the network coverage rate can still be improved. Furthermore, the energy consumption balance of the network formed by CDA performs poorly when the number of nodes is relatively sparse. On the other hand, some studies [27,28] have also investigated network cluster formation in wireless sensor networks (WSNs). Heinzelman *et al.* [29] proposed the low-energy adaptive clustering hierarchy (LEACH) algorithm. This algorithm selects a cluster head node randomly and completes clustering with the principle of the nearest neighbor. Its operation is simple, but it cannot control the distribution of the cluster head node effectively. Mao *et al.* [30] proposed an energy-efficient clustering scheme (EECS), which selects a cluster head node according to the residual energy of the node and then finishes the network cluster. Compared to LEACH, EECS selects the nodes with more residual energy preferentially as cluster head nodes, which reduces the exchange frequency of the cluster head node and enhances the network stability. Tsai [31] proposed a coverage-preserving routing protocol for WSNs. It selects the cluster head node based on the coverage redundancy rate of the node and then finishes the network cluster. Consequently, the network operates based on the cluster, and the great network coverage rate is maintained. In the method, a node having a greater coverage redundancy rate has priority to become a cluster head node, which reduces the node failure impact on the network coverage rate and slows down the drop speed of the network coverage rate. Abbasi *et al.* [32] surveyed different cluster formation algorithms, analyzing their objects, features, *etc.* Lloret *et al.* [33] proposed an algorithm that can structure the topology of different WSNs to coexist in the same environment based on network clustering, where cluster head nodes manage their own networks and have connections with other cluster head nodes, resulting in good network connectivity and scalability of the whole parallel network structure. Yuan *et al.* [34] proposed an uneven cluster mechanism. The mechanism divides the network into many clusters with different sizes. Among them, a cluster, further from the sink node, has a greater size. On the contrary, a cluster, closer to the sink node has a smaller size. The cluster distribution may balance the network energy consumption and extend the network lifetime. Qiao *et al.* [35] proposed a chain structure-based uneven cluster routing algorithm. It establishes a dynamic multiple-hop route considering the uneven cluster mechanism for transmitting data, which can balance the energy consumption of nodes and prolong the network lifetime.

To solve the aforementioned problems on move-restricted node self-deployment and according to the aforementioned cluster formulation algorithm description, this study proposes the uneven cluster and radius-adjusting self-deployment algorithm (URSA). After nodes are scattered on the water surface randomly and uniformly, each node begins the uneven clustering process according to the distance to the sink node. The cluster nodes then use the hybrid radius path-selection method simultaneously to form a connected path to the sink node. In the depth-adjustment phase, the cluster head nodes adjust their own depths according to the principle of maintaining the layout formed on the water surface by the uneven clustering process. For each of its in-cluster nodes, the cluster head node calculates some corresponding coverage redundancy rates (*CRR*) by assuming that the in-cluster node is deployed on each position within the maximum communication radius of its basic nodes (*i.e.*, nodes that belong to the same cluster head node and that is the next-hop of the in-cluster node). The cluster head node subsequently minimizes the hop number of the in-cluster node to optimize and adjust its position. This algorithm forms a network layout of uneven distribution. In the distribution, the node density and number of cluster head nodes of the area close to the sink node increase; the scale of the cluster in that area becomes small. As a result, the number of nodes near the sink node then increases, and the network reliability improves. The process of optimizing and adjusting the position of the in-cluster nodes further improves the network coverage rate, decreases the hop of in-cluster nodes and decreases the energy consumption of network deployment. The connected topological structure formed during the process of node deployment (*i.e.,* the cluster head node farther from the sink node has a greater communication radius, and the cluster farther from the sink node has a larger scale) also balances the energy consumption of network operation. The simulation results show that compared to CDA having a good deployment performance, URSA can improve network reliability, balances and reduces network energy consumption and improves the network coverage rate.

The rest of this paper is organized as follows. Section 2 describes the system model, assumptions and definitions considered in this study. Section 3 presents the details of URSA. Section 4 analyzes the complexity of URSA. Section 5 discusses the performance study and provides a detailed analysis of its result. Finally, Section 6 concludes the paper and plans some future works.

## 2. Models and Definitions

### 2.1. Network Model

**Figure 1:**
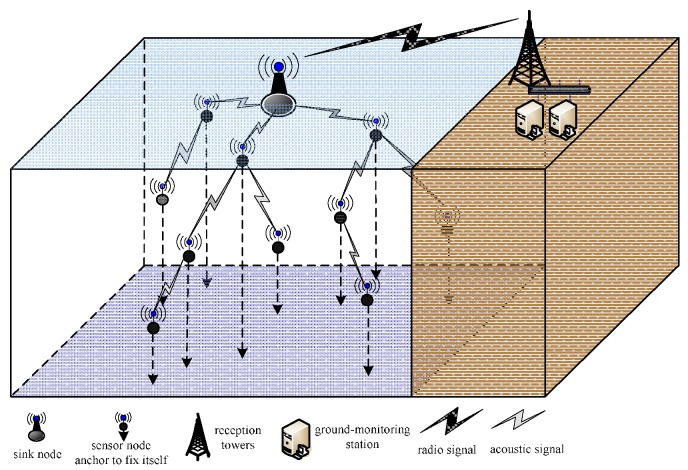
Underwater wireless sensor network (UWSN) system model.

Assume that *N* sensor nodes are scattered on the water surface randomly and uniformly and are floating on the water surface with buoys. Each node has communication, perception and mobility capabilities (perpendicular to the horizontal direction), and the positions of the sensor nodes are adjusted underwater by moving them to form a 3D coverage network. A typical UWSN architecture is depicted in Figure 1. In this model, the sink node communicates with the ground-monitoring station by radio; nodes communicate with one another by acoustic channels and maintain connectivity with the sink node via one- or multi-hop paths. Furthermore, these nodes anchor to fix their position once they have adjusted their depth. This study denotes the *i*-th node by *s_i_*, and the corresponding node set *S =* {*s_1_*, *s_2_*, … *s_n_*}. The following assumptions are considered:

1. The Boolean perception model is adopted to describe the node sensing. If the sensing radius of the node *s_i_* is *R_s_*, the space sensed by the node is a sphere whose center is the node location and *R_s_* as the radius. An example is shown in Figure 2. The sphere with radius *R_s_* is the sensed space of *s_i_*. *P_1_* is within the sphere and can be covered by *s_i_*. On the contrary, *P_2_* is beyond the sphere and cannot be covered by *s_i_*.
  **Figure 2:**
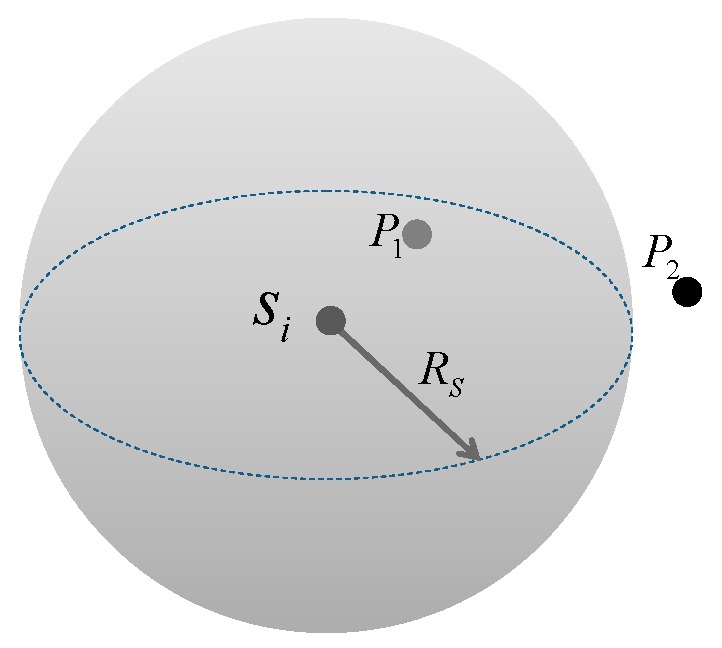
3D Boolean perception model.
2. All nodes are isomorphic before deployment, but can adjust their transmission power by themselves, *i.e.*, the communication radius *R_c_* of the node can be adjusted with an adjusting precision of 1 m, but not more than the maximum communication radius *R_c__Max* determined by the physical device. The communication radius of a node will be changed by the distance to its next-hop node, *i.e.*, the communication radius of a node is the rounded-down value of the distance between them when its next-hop node is determined.
3. The network has only one sink node. Its position is fixed at the center of the water, and the transmission power and energy can be infinite.
4. A node knows its own position and can determine the distance to the source according to the strength of the received signal.

### 2.2. Node Energy Consumption Model

Underwater sensor network nodes communicate with one another by acoustic signals. Accordingly, this study uses an energy consumption model of underwater sensor network data communication with the sound wave as the medium [36]. The underwater acoustic signal attenuation model *A*(*d*) is given as follows:

(1) $$A\left(d\left(s_{se},s_{rec}\right)\right)=d^{\lambda }\left(s_{se},s_{rec}\right)\alpha^{d\left(s_{se},s_{rec}\right)}$$

Equation (1) describes the energy attenuation when the data packet transmitting distance from the source node *s_se_* to the destination node *s_rec_* is *d*(*s_se_*, *s_rec_*), where *λ* is the energy diffusion factor (the cylindrical diffusion is one; the actual situation is 1.5; and the spherical diffusion is two). The parameter *α =* 10*^a^*^(^$F_{r}$^)/10^, where the absorption coefficient *a*(*F_r_*) is shown as follows:

(2) $$a(F_{r})=0.11\frac{10^{-3}F_{r}^{2}}{1+F_{r}^{2}}+44\frac{10^{-3}F_{r}^{2}}{4100+F_{r}^{2}}+2.75\times 10^{-7}F_{r}^{2}+3\times 10^{-6}$$

where *F_r_* is the carrier frequency, with the unit of kHz. The unit of the absorption coefficient is dB/m.

The energy for the nodes to send data *E_tx_*(*d*(*s_se_*,*s_rec_*)) is expressed as follows:

(3) $$E_{tx}\left(d\left(s_{se},s_{rec}\right)\right)=P_{r}\times T_{p}\times A\left(d\left(s_{se},s_{rec}\right)\right)$$

where *T_p_* is the data transmission time and *P_r_* is the minimum power packets that can be received.

### 2.3. Coverage Redundancy Rate

The coverage redundancy rate (*CRR*) of node *s_i_* is defined as the ratio of the sensing overlapping area of itself with other nodes within its one-hop communication radius and its sensing area [37]. γ(*s_i_*) is formulated as follows:

(4) $$\gamma (s_{i})=\frac{volume\left(\left(\underset{s_{j}\in neighbor(s_{i})}{\cup }\;area(s_{j})\right)\cap area(s_{i})\right)}{volume\left(area(s_{i})\right)}$$

where *area*(*s_i_*) represents the sensing area of node *s_i_*.

The formulation in practical use is defined as follows:

(5) $$\gamma (s_{i})=1-\prod_{j=1}^{n}\left(1-\frac{2\left[\frac{2}{3}\pi \left(R_{s}^{3}-\frac{d^{3}(s_{i},s_{j})}{8}\right)-\frac{\pi d(s_{i},s_{j})}{2}\left(R_{s}^{2}-\frac{d^{2}(s_{i},s_{j})}{4}\right)\right]}{\frac{4}{3}\pi R_{s}^{3}}\right)$$

where *n* is the number of neighbor nodes of node *s_i_* and *d*(*s_i_,s_j_*) is the distance between node *s_i_* and its neighbor node *s_j_*.

### 2.4. Network Coverage Rate

The network coverage rate, which reflects the coverage degree of an underwater sensor network covering a monitoring area or targets, is also a primary standard to evaluate a node-deployment algorithm. This standard is defined as the ratio of the effective area, the number of UWSN covers of the targets to the entire area of the target region or all target numbers by using the *Cor* expression; the formula is expressed as follows [26]:

(6) $$Cor=\frac{V_{\mathrm{cov}ered}}{V_{region}}$$

where *V_covered_* is the volume of the monitoring area covered by active nodes and *V_region_* is the volume of the total monitoring area.

### 2.5. Network Connectivity Rate

The network connectivity rate is an important criterion of the service quality of sensor networks and the premise of sensor network application. This criterion refers to the ratio of the number of nodes that connect with the sink node by one or several hops to the total number of nodes by using the *Cer* expression; the formulation is expressed as follows [26]:

(7) $$Cer=\frac{N_{connection}}{N_{all}}$$

where *N_connection_* is the number of nodes connected to the sink node and *N_all_* is the total number of nodes in the UWSN.

### 2.6. Network Reliability

In UWSNs, network reliability reflects the effect on the network service quality when a node does not function because of serious environmental and external factors or a lack of energy. From node deployment, network reliability can be expressed by the redundancy of a network node. In this study, network reliability can be expressed by the number of nodes within a certain range of the sink node and the average number of their neighbor nodes. If the two indicators are great, the network reliability is also great.

## 3. Algorithm Description and Process

### 3.1. Problem Description

Some scholars have already researched the node self-deployment problem for move-restricted underwater sensor nodes. However, these algorithms are suitable only for the node deployment of underwater sensor nodes with a fixed communication radius and only consider the criterion of network coverage or the network connectivity rate without considering the network reliability problem and the energy consumption balance of the network. In existing algorithms, adjacent nodes are far away from one another as much as possible to minimize the overlapping area among themselves and to maximize the network coverage rate. Consequently, the probability that nodes are deployed near the sink node decreases to some degree, and the number of nodes within a certain range around the sink node cannot be guaranteed. Even the use of a topology control method with a good effect cannot improve network performance after deployment. In addition, in the process of adjusting the node depth, algorithms only focus on the *CRR* of the position wherein the overlapping area of one node with a specific node is the smallest and ignores the *CRR* of the position where the overlapping area is not the smallest. Therefore, the network coverage rate should be improved. Moreover, existing algorithms that consider the network connectivity rate (*i.e.,* CDA) do not consider the balance of network energy consumption during network operations. When the number of nodes is dense, the network formed by CDA can modify the network topology by a routing protocol to achieve energy consumption balance. By contrast, when the number of nodes is relatively sparse, the network formed by CDA may have no other connected topology or ineffectively route protocols because the number of nodes is insufficient. At this time, an operated network can only use the topological structure formed during the process of node deployment. Furthermore, the energy consumption balance of the topological structure performs badly, *i.e.*, the energy consumption balance of the network formed by CDA is poor when the number of nodes is relatively sparse. Therefore, this study defines the following problem: “After *N* nodes are randomly and uniformly scattered on the water surface of the target area, a node self-deployment algorithm with an adjustable communication radius is designed to adjust the depth of nodes, improve network reliability, balance, reduce energy consumption, and maximize network coverage on the premise of maintaining network connectivity”.

To solve the defined problem, this study proposes a distributed self-deployment algorithm for underwater sensor nodes (*i.e*., URSA). After all nodes are scattered on the water surface randomly and uniformly, each node firstly begins the process of uneven clustering according to the distance to the sink node. This process forms a layout wherein the number of cluster head nodes in an area close to the sink node becomes large and the scale of the cluster in that area becomes small. Second, every cluster head node uses the hybrid radius path-selection method to select its next-hop node concurrently according to the principle of minimizing the energy consumption of sending a message from itself to the sink node. The cluster head node then forms a connected path to the sink node. The feature of the connected path is that if the cluster head node is far away from the sink node, its path to the next-hop node is long, thus ensuring network connectivity and saving and balancing energy consumption in network operations. Third, each cluster begins to adjust the depth in an iterative manner, and the corresponding cluster head node selects its own adjusting position with the principle that the distance between itself and its next-hop node after adjustment is 1 m longer than that before adjustment. This process retains the layout of the uneven cluster (*i.e.,* the number of cluster head nodes in an area close to the sink node increases, whereas the communication radius of the cluster head node in that area decreases), increases the node number within the scope of the sink node, improves the network reliability and balances network energy consumption. Finally, for each in-cluster node, the cluster head node calculates some corresponding *CRR* rates by supposing that the in-cluster node is deployed on each position within the maximum communication radius of its basic nodes and selects the best position for every in-cluster node by the priority of the hop of the basic node of the in-cluster node on the basis of decreasing the network coverage rate. This process decreases the hop number of the in-cluster nodes, increases the chances of the nodes near the sink node, decreases the energy consumption of deployment and improves the total coverage rate. The detailed description is presented as follows.

### 3.2. Algorithm Description

URSA is divided into the following four steps: (1) uneven clustering [38]; (2) constructing a connected path by the hybrid radius path-selection method; (3) the cluster head node calculating the depth of each node in a cluster; and (4) finding a next cluster needing to be adjusted. The detailed steps of the algorithm are as follows.

#### 3.2.1. Uneven Clustering

After nodes are scattered on the water surface randomly and uniformly, the sink node broadcasts to all nodes. Every node *s_i_* calculates the distance to the sink node *d*(*s_i_,Sink*) according to the strength of the signal it receives and sets its probability threshold *T_h_*(*s_i_*) by Equation (8). Every node *s_i_* then generates a random number *r*(*s_i_*) from zero to one and compares this number to *T_h_*(*s_i_*). If *r*(*s_i_*) *< T_h_*(*s_i_*), node *s_i_* becomes a provisional cluster head node and joins the set of provisional cluster head nodes *P* (*P =* {*p_1_,p_2_,…p_n1_*} *=* {*s_i_| r*(*s_i_*) *< T_h_*(*s_i_*)*, s_i_* ϵ *S*}, where *p_i_* represents the *i*-th provisional cluster head node); otherwise, node *s_i_* exits from the cluster competition and transforms into sleep mode.

(8) $$T_{h}(s_{i})=\left\{ \begin{array}{cc} T_{h1} & d(s_{i},Sink)<d_{hot}\\ T_{h2} & d_{hot}\leq d(s_{i},Sink)<2d_{hot}\\ T_{h3} & d(s_{i},Sink)\geq 2d_{hot} \end{array}\right.$$

where *T_h1_*, *T_h2_* and *T_h3_* are the probability threshold parameters, and *T_h1_* > *T_h2_* > *T_h3_*. *d_hot_* represents the radius of the hot-spot area. Equation (8) represents that if a node is close to the sink node, its *T_h_* is great. In other words, the nodes near the sink node have a great probability to become a provisional cluster head node, whose aim is to increase the number of the final cluster head nodes near the sink node as much as possible.

Every provisional cluster head node *p_i_* defines its own competition radius *R*(*p_i_*) by Equation (9) and broadcasts its status message (node ID, *d*(*p_i_,Sink*), *R*(*p_i_*)) with the communication radius *R_0_*. After a provisional cluster head node *p_i_* receives the message, it calculates the distance *d*(*p_i_*, *p_j_*) to the signal-sending node *p_j_* and defines the set of its own competition neighbor nodes *S_CH_*(*p_i_*) by Equation (10), namely the provisional cluster head node within its competition radius *R*(*p_i_*).

(9) $$R(p_{i})=\left(1-c\frac{d_{\max}-d(p_{i},Sink)}{d_{\max}}\right)R_{0}$$

where *d_max_* is the farthest distance between any points in the water surface of the target area and the sink node; *c* is the proportional constant; *R_0_* is the maximum competition radius.

(10) $$S_{CH}\left(p_{i}\right)=\left\{p_{j}|d(p_{i},p_{j})\leq \max\left(R(p_{i}),R(p_{j})\right),d(p_{i},p_{j})\leq R_{0}\right\}$$

In the stage of cluster head competition, all provisional cluster head nodes set the same waiting time (*Time*). During the *Time*, each provisional cluster head node *p_i_* compares its distance to the sink node with the nodes in *S_CH_*(*p_i_*) and determines whether it will become a final cluster head node (*i.e.,* cluster head node) *f_m_* (*f_m_* is the *m*-th element in the set of the final cluster head node, namely the *m*-th final cluster head node) according to the following conditions:

(1) *d*(*p_i_,Sink*) ≤ min(*d*(*p_j_,Sink*))*, p_j_* ϵ *S_CH_*(*p_i_*): *p_i_* becomes *f_m_* and broadcasts the “been cluster head” message *M_CH_* to notify its competition neighbor nodes with the communication radius of *R_0_.*
(2) *d*(*p_i_,Sink*) > min(*d*(*p_j_,Sink*))*, p_j_* ϵ *S_CH_*(*p_i_*): *p_i_* waits for messages from its competition neighbor nodes and takes the following corresponding operation according to the messages it receives:
  ➢ *p_i_* receives *M_CH_* broadcasted by one of its competition neighbor node *p_j_*, gives up the cluster head competition and broadcasts the “exit competition” message *M_GC_*. It then converts to sleep mode.
  ➢ *p_i_* receives *M_GC_* broadcasted by one of its competition neighbor node *p_j_*, removes node *p_j_* from *S_CH_*(*p_i_*), then compares the distance to the sink node with its updating competition neighbor nodes and continues the preceding operations.
  ➢ *p_i_* cannot receive any message in *Time* and becomes *f_m_*.

After *Time* is over, every cluster head node *f_m_* broadcasts the “elect cluster head” message (the ID of *f_m_*) with the maximum communication radius *R_c__Max* to stimulate the non-cluster head nodes to join one of the clusters and the cluster head node to save the information of its neighbor cluster head nodes. When the cluster head node *f_j_* receives that message, it saves the distance *d*(*f_m_*,*f_j_*) and places the ID of *f_m_* into the set of its neighbor cluster head nodes *N_CH_*(*f_j_*); when the non-cluster head node receives that message, it calculates the distance to every cluster head who is the source of that message and replies with the “join” message (non-cluster head node ID) to its nearest cluster head node. For the non-cluster head nodes that do not receive any message, they broadcast with *R_c__Max* and join themselves into the nearest cluster according to the reply of their neighbor nodes. They transmit the “join” message to the cluster head node by using the neighbor node as the intermediate node.

**Figure 3:**
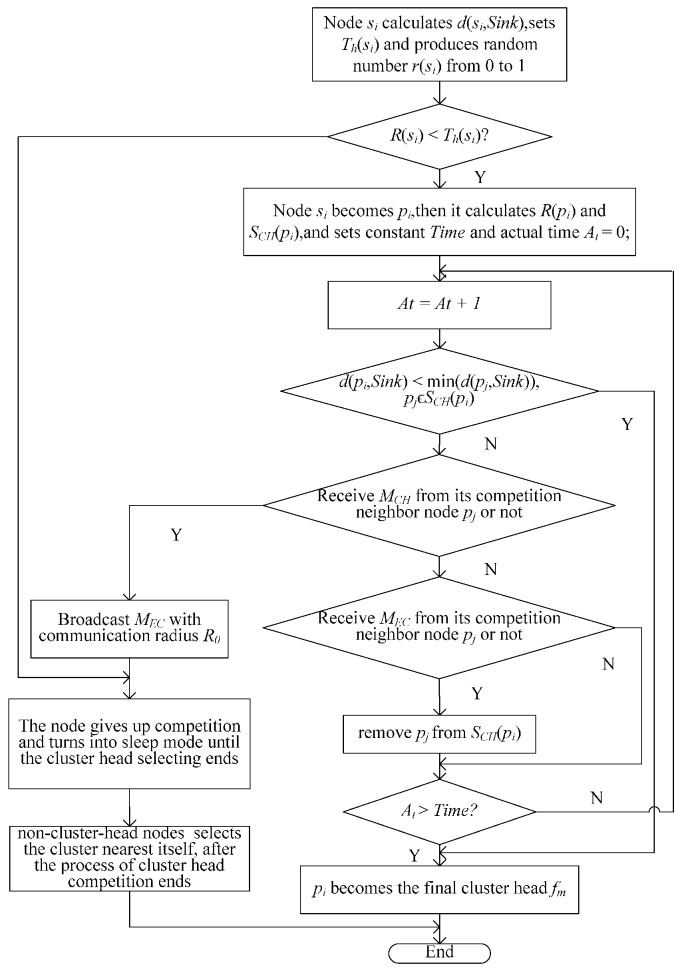
Flow chart of Section 3.2.1.

The flow chart of Section 3.2.1 is shown in Figure 3 with node *s_i_* as an example.

#### 3.2.2. Constructing a Connected Path by the Hybrid Radius Path-Selection Method

When every cluster head node *f_m_* does not receive the reply of non-cluster head nodes over a period of time, *f_m_* will construct a connected path to the sink node. *f_m_* first compares *d*(*f_m_*,*Sink*) to the radius of the hot-spot area *d_hot_*. If *d*(*f_m_*,*Sink*) *< d_hot_*, *f_m_* connects with the sink node directly; otherwise, *f_i_* will find its next-hop node from the set of its neighbor cluster head nodes *N_CH_*(*f_m_*). The detailed process is expressed as follows:

(1) *f_m_* selects the cluster head nodes in the forward direction (*i.e.,* the direction close to the sink node) from the nodes in *N_CH_*(*f_m_*) by Equation (11). These nodes are denoted by the cluster head nodes of the forward direction of *f_m_ B_CH_*(*f_m_*):
  (11) $$B_{CH}(f_{m})=\left\{f_{j}|d(f_{j},Sink)<d(f_{m},Sink),f_{j}\in N_{CH}(f_{m})\right\}$$
(2) *f_m_* selects the *k* nodes nearest to itself from the nodes in *B_CH_*(*f_m_*) as the set of alternative next-hop nodes *F_CH_*(*f_m_*). If the number of the nodes in *B_CH_*(*f_m_*) is smaller than *k*, *F_CH_*(*f_m_*) = *B­_CH_*(*f_m_*). If the node number is zero, Step (3) is performed; otherwise, Step (4) is performed.
(3) *f_m_* selects *k* non-cluster head nodes whose cluster head nodes are not *f_m_* in the forward direction within its *R_c__Max* as the set of alternative next-hop nodes *F_CH_*(*f_m_*). This process guarantees that the communication radius of *f_m_* is greater than the communication radius of its next-hop node.
(4) *f_m_* selects a node *s_j_* from the nodes in *F_CH_*(*f_m_*) by minimizing the sum of the distance to *f_m_* and the distance to the sink node as its next-hop node *next*(*f_m_*):
  (12) $$next(f_{m})=\left\{s_{j}|\min\left(d(f_{m},s_{j})+d(s_{j},Sink)\right),s_{j}\in F_{CH}\left(f_{m}\right)\right\}$$
(5) If *next*(*f_m_*) is a non-cluster head node, the node becomes a cluster head node and finds its next-hop node according to the preceding process. Otherwise, the entire process ends.

#### 3.2.3. Cluster Head Node Calculating the Depth of Each Node in a Cluster

When one cluster head node *f_m_* receives the “calculate diving depth” message *M_CD_*, *f_m_* optimizes its own position and the position of its in-cluster nodes. After the depths of all nodes in its cluster are calculated, each node dives to its position synchronously.

*f_m_* initially calculates its own diving position according to the principle of maintaining and adjusting the layout on the water surface formed by uneven clustering. The rules are as follows:

(1) *f_m_* is a common node (having both the last-hop node and the next-hop node). If the communication radius of *f_m_* on the water surface *R_UA_*(*f_m_*) plus one is greater than the adjusted communication radius *R_A_*(*f_j_*) of its next-hop node *f_j_*, *i.e., f_m_* satisfies the feature of the uneven clustering after it adjusts its depth, *f_m_* regards its adjusted communication radius as *R_UA_*(*f_m_*) *+* 1 and calculates its diving depth; otherwise, *f_m_* regards the adjusted communication radius of its next-hop node *R_A_*(*f_j_*) plus one as its adjusted communication radius and calculates its diving depth. This process of calculating the diving depth of *f_m_ dep*(*f_m_*) is formulated as follows:
  (13) $$dep(f_{m})=dep(f_{j})+\left\{ \begin{array}{cc} \sqrt{2R_{UA}(f_{m})+1} & R_{UA}(f_{m})+1>R_{A}(f_{j})\\ \sqrt{{\left(R_{A}(f_{j})+1\right)}^{2}-{\left(R_{UA}(f_{m})\right)}^{2}} & R_{UA}(f_{m})+1\leq R_{UA}(f_{j}) \end{array}\right.$$
  where *R_UA_*(*f_m_*) is the communication radius of *f_m_* before it adjusts its depth. In other words, *R_UA_*(*f_m_*) represents the distance between itself and its next-hop node on the water surface. *R_A_*(*f_m_*) is the communication radius of *f_m_* after it adjusts its depth. In other words, *R_A_*(*f_m_*) represents the distance between itself and its next-hop node after *f_m_* adjusts its depth.
(2) If *f_m_* is a leaf node (having only next-hop nodes), it can reduce the *CRR* with its last-hop node by increasing its own depth. *f_m_* determines its depth *dep*(*f_m_*) by Equation (14) on the basis of whether it is the one-hop node of the sink node.

(14) $$dep\left(f_{m}\right)=dep\left(f_{j}\right)+\left\{ \begin{array}{cc} \sqrt{d_{hot}^{2}-d^{2}\left(f_{m},Sink\right)} & d\left(f_{m},Sink\right)\leq d_{hot}\\ \sqrt{{\left(2R_{s}\right)}^{2}-{\left(R_{UA}(f_{m})\right)}^{2}} & d\left(f_{m},Sink\right)>d_{hot} \end{array}\right.$$

After the cluster head node *f_m_* defines its depth, it updates the node set *all_deployed* consisting of nodes that have adjusted their depths in the entire network and the node set *part_deployed* consisting of nodes with depths calculated in the cluster and begins to calculate the depth of its in-cluster nodes. The cluster head node *f_m_* defines the depth of one in-cluster node *N_f_* every round. In each round, *f_m_* initially selects each node in order from the nodes in *part_deployed* as the basic node *b* of *N_f_* (*i.e.,* nodes in the same cluster with *N_f_*, as the next-hop nodes of *N_f_*). For every basic node *b*, *f_m_* individually calculates the diving depth of *N_f_* by Equations (15) and (16) when the distance *d_adjust* between the adjusted position of *N_f_* and the adjusted position of *b* is *R_c__Max*, *R_c__Max-1*…*d*(*N_f_*,*b*). *f_m_* then selects the minimum *CRR* as the *CRR* of basic node *b* and saves the corresponding depth (when the minimum *CRR* corresponds to several different depths, *f_m_* selects the corresponding depth by the communication radius of node *N_f_* and the diving distance of *N_f_* in order).

(15) $$dis\_vertical=\sqrt{d\_adjust^{2}-d^{2}(N_{f},b)}$$

(16) $$dep(N_{f})=\left\{ \begin{array}{cc} R_{s} & dep(b)-dis\_vertical<R_{s}\\ Depth-R_{s} & dep(b)+dis\_vertical>Depth-R_{s}\\ dep(b)\pm dis\_vertical & others \end{array}\right.$$

where *dis_vertical* is the vertical distance between the adjusted position of *N_f_* and the adjusted position of *b*; *Depth* is the depth of the target area. Equation (16) represents the diving depth of *N_f_* when the diving depth of *N_f_* is greater than *Depth*-*R_s_*_­_ or smaller than *R_s_*. In other words, at that moment, some useless region exists in the sensing area of *N_f_*, and the diving depth of *N_f_* is equal to *Depth*-*R_s_* or *R_s_*.

*f_m_* subsequently compares the *CRR* of every basic node *b* to select the minimum *CRR*. Thereafter, *f_m_* compares the hop number of every basic node to the *CRR* that is within the *rang* range of the minimum *CRR* and selects the basic node with the minimum hop number as the basic node of *N_f_* (when the hop number is equal, *f_m_* selects it by the communication radius and diving depth in order of node *N_f_*). *f_m_* determines the optimal position of node *N_f_*. *f_m_* finally updates *part_deployed* and *all_deployed*.

**Figure 4:**
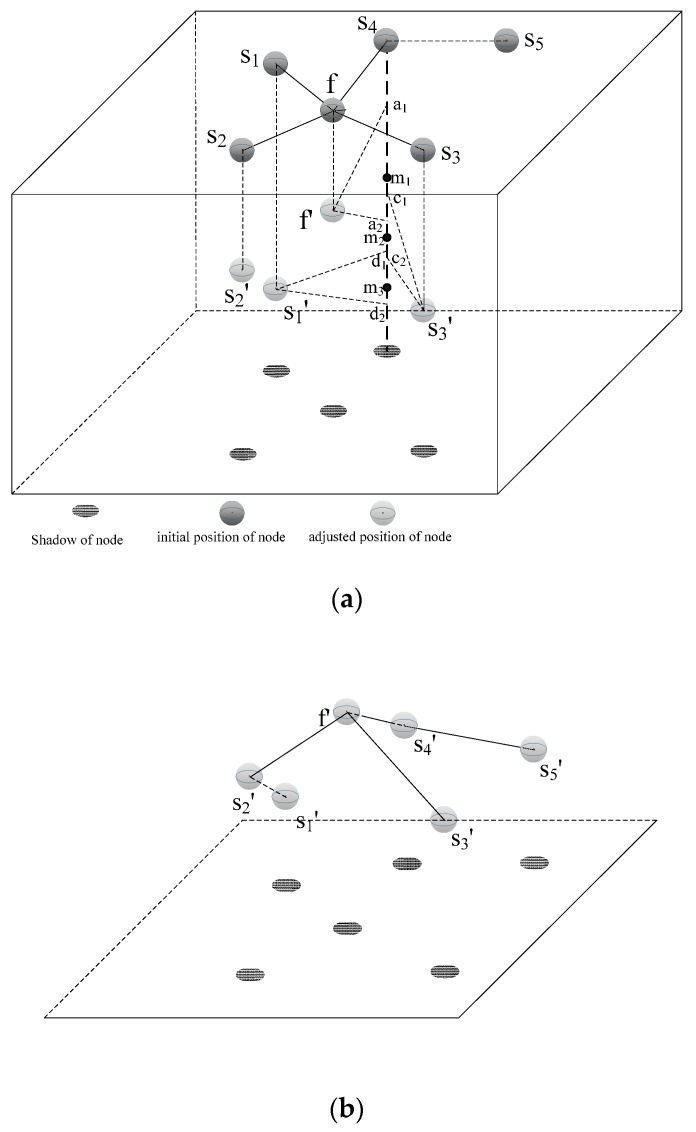
(**a**) Depth calculation process in a cluster (assume that the *CRR* on *m_3_* is the minimum and the *CRR* on *m_1_* is within *rang* range of the minimum *CRR*; the basic node (next-hop node) of *s_2_* and *s_3_* is *f*, and the basic node of *s_1_* is *s_2_*, after their depth is already calculated); (**b**) 3D result of nodes’ depth adjusting.

An example for the process by which the cluster head node calculates the depths of its in-cluster nodes is described in Figure 4a. In the figure, *s_1_*, *s_2_*, *s_3_*, *s_4_*, *s_5_* and *f* are on the water surface. Among them, *f* represents a cluster head node, and others represent in-cluster nodes. In addition, *s_1_*, *s_2_*, *s_3_* and *f* are in the *part_deployed*. In other words, their depths have been already calculated in the cluster. When *f* calculates the depth of *s_5_*, it finds that the distance between *s_5_* and each node in *part_deployed* is greater than *R_c__Max*. Thus, it ignores calculating the depth of *s_5_* tentatively and continues to calculate the depth of another node (*i.e.*, *s_4_*). It firstly selects *s_1_* as the basic node of *s_4_* and calculates the possible diving depth assuming that the distance between the adjusted position of *s_4_* and *s_1_'* is respectively *R_c__Max*, *R_c__Max-1*…*d*(*s_4_*,*s_1_*). Consequently, all of the possible diving depths are on the line segment *d_1_d_2_*. It then calculates the *CRR* of every position on *d_1_d_2_* and chooses the minimum *CRR* as the *CRR* of *s_1_* and saves the corresponding position *m_3_*. In a similar way, all of the possible diving depths are on the line segment *c_1_c_2_*, and the position of the minimum *CRR* is *m_2_*, when it selects the *s_3_* as the basic node of *s_4_*; all of the possible diving depths are on the line segment *a_1_a_2_*, and the position of the minimum *CRR* is *m_1_*, when it selects the *f.* Specifically, when it selects the *s_2_* as the basic node of *s_4_*, it ignores the situation because the distance between *s_4_* and *s_2_* is greater than *R_c__Max*. Finally, *f* finds the minimum *CRR* among these position (*i.e.*, *m_1_*, *m_2_*, *m_3_*) and compares the hop of the basic node whose *CRR* is within *rang* range of the minimum *CRR* (*i.e*., compares the hop of *s_1_* and *f*). Consequently, *f* selects *m_1_* as the diving depth of *s_4_* and *f* as the basic node of *s_4_*. After finishing calculating the depth of *s_4_*, it calculates *s_5_* again. At last, each node synchronously dives to the position according to the calculation result. The result of the nodes’ depth adjusting is depicted in Figure 4b.

**Figure 5:**
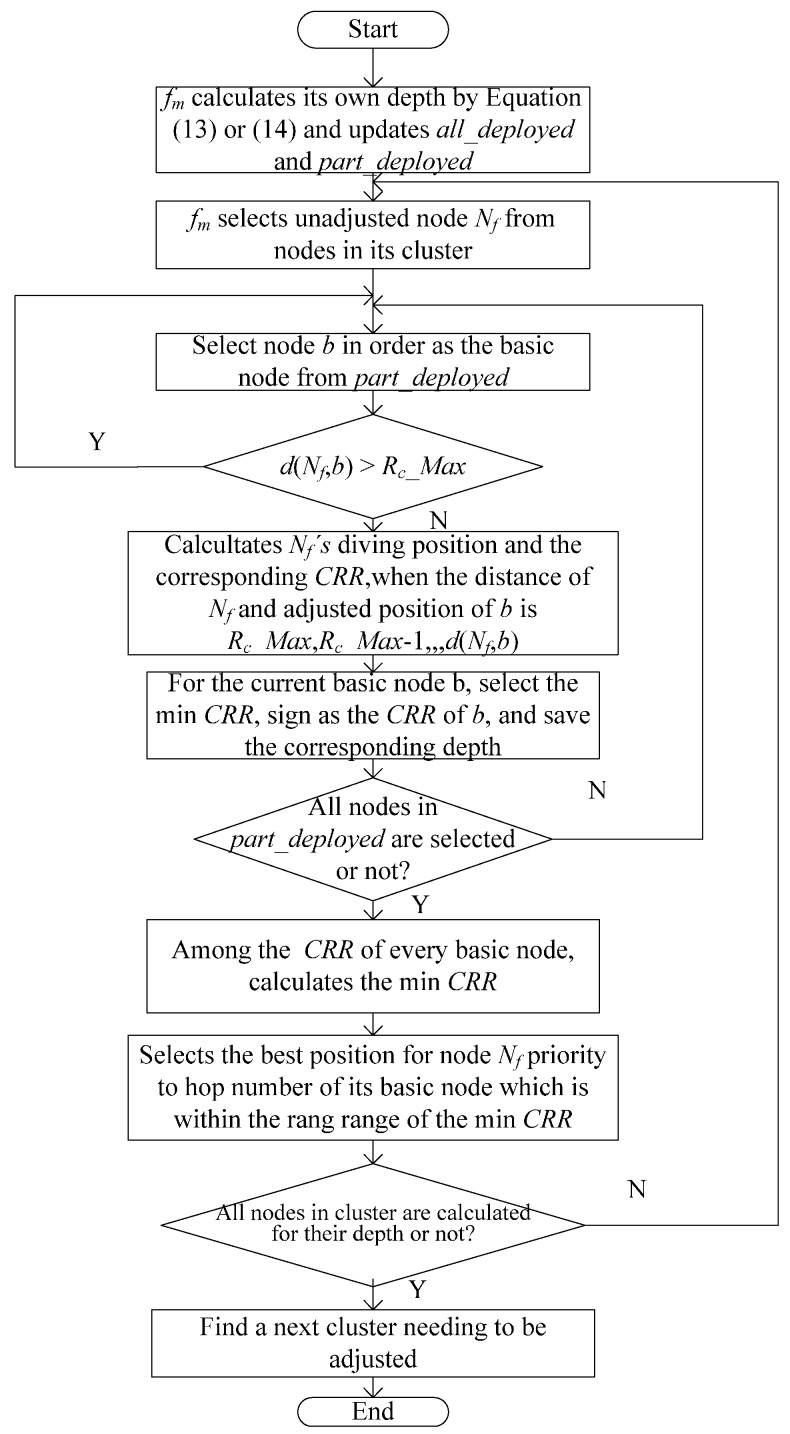
Flow chart of Section 3.2.3.

The flow chart of Section 3.2.3 is shown in Figure 5 with a cluster head node *f_m_* as an example.

#### 3.2.4. Finding a Next Cluster Needing to Adjust

After the cluster head node *f_m_* calculates the diving depth of all nodes in its cluster, it transmits the “adjust depth” message (diving depth, basic node ID) to the corresponding in-cluster node to notify them to dive. After all nodes dive to the specified location, *f_m_* broadcasts the “update” message (ID, diving depth and adjusted communication radius of every node), and the other cluster head nodes that receive the message update their *all_deployed*. *f_m_* then queries its last-hop cluster head nodes whether some cluster head nodes have not adjusted. Thus, *f_m_* transmits *M_CD_* to one of them, and the cluster head node receiving the message operates by Section 3.2.3. Otherwise, *f_m_* transmits the “been adjusted” message *M_AD_* to its next-hop *f_j_*, and *f_j_* selects to transmit *M_CD_* or *M_AD_* according to the situation of its last-hop cluster head node. This process is repeated until all clusters are adjusted.

## 4. Algorithm Analysis

### 4.1. Message Flow between Nodes

In URSA, message flow between nodes takes place during the process of the uneven clustering, constructing a connected path and the position adjustment of in-cluster nodes. This paper illustrates the message flow between nodes in the algorithm based on some specific nodes.

#### 4.1.1. Message Flow during Uneven Clustering

**Figure 6:**
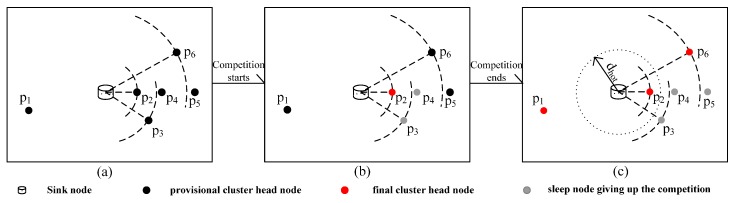
Several stage statuses of uneven clustering. (**a**) Initialization phase of cluster head node competition; (**b**) Phase of cluster head node competition; (**c**) End of cluster head node competition. (Assume that *p_2_*, *p_3_* and *p_4_* can communicate with each other within *R_0_* and that they are the competition neighbor nodes for each other; so do *p_4_*, *p_5_* and *p_6_*. In addition, *p_2_* is the next-hop node of *p_6_*, and the distance between *p_1_* and the sink node is greater than *R_c__Max*).

There is message flow in the several stages of the uneven clustering, as shown in Figure 6.

**Figure 7:**
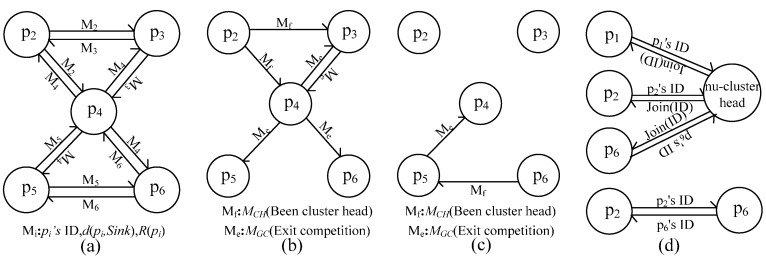
(**a**) Message flow in the initialization phase of cluster head node competition; (**b**,**c**) message flow at the stage of cluster head node competition; (**d**) message flow after cluster head node competition.

In Figure 6a, the nodes are in the initialization phase of cluster head node competition. At this time, every provisional cluster head node broadcasts within *R_­0_*; the process of message flow between nodes is shown in Figure 7a. Then, entering into the phrase of the cluster head competition is shown in Figure 6b. *p_2_* firstly becomes a final cluster head node and broadcasts *M_CH_* within *R_0_*, and *p_3_*, as well as *p_4_* exit the competition due to receiving *M_CH_* from *p_2_* and broadcast *M_GC_*, respectively, which is shown in Figure 7b. Afterwards, *p_5_* and *p_6_* respectively remove *p_4_*. At the same time, *p_6_* becomes a final cluster head node, and *p_5_* exits the competition; the message flow is shown in Figure 7c. After the cluster head node competition, shown in Figure 6c, the final cluster head nodes broadcast their ID, and the non-cluster head nodes reply with their ID to the corresponding cluster head node, the message flow of which is shown in Figure 7d.

#### 4.1.2. Message Flow during Path Selection

**Figure 8:**
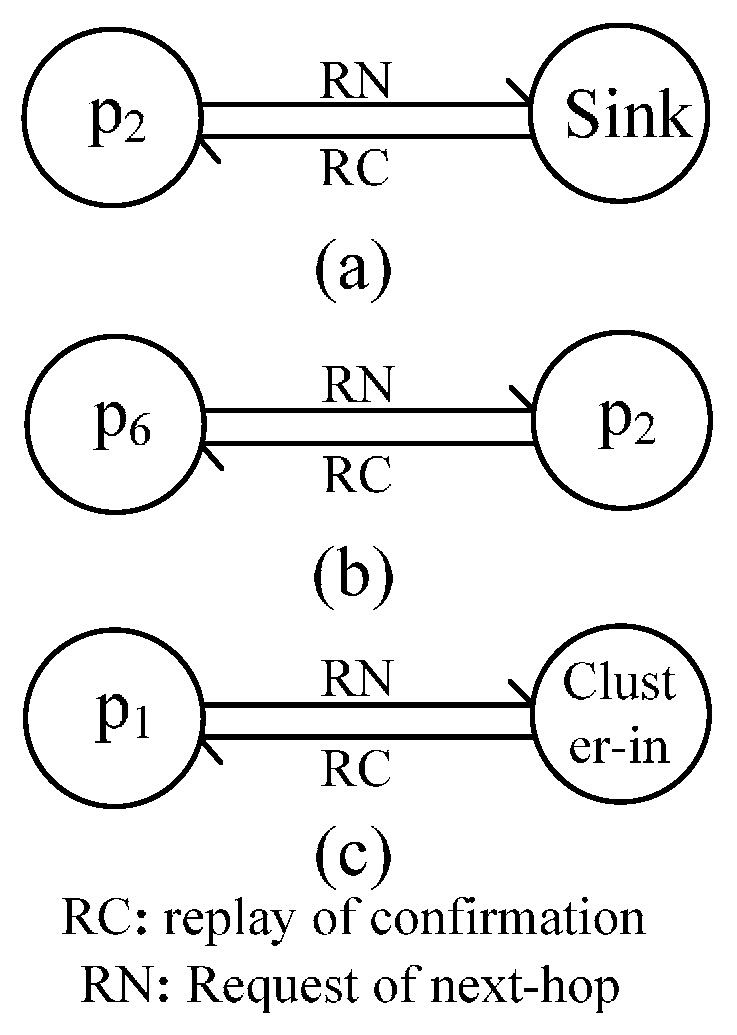
Message flow during path selection. (**a**) Message flow with Sink node; (**b**) Message flow with cluster head node; (**c**) Message flow with non-cluster node.

After clustering, cluster head nodes begin to build a connected path. When a node’s distance to the sink node is less than *d_hot_*, such as *p_2_* in Figure 6c, the node sends a request message to the sink node, and the sink node replies with a confirmation message to the node; the process is shown in Figure 8a. When a node’s distance to the sink node is greater than *d_hot_* and there are other cluster head nodes within its *R_c__max*, such as *p_6_* in Figure 6c, it has a message exchange with a cluster head node, which is shown in Figure 8b. In a similar way, when a cluster head node has no neighbors within its *R_c__max*, such as *p_1_* in Figure 6c, it has a message exchange with a non-cluster head node; the message flow between them is shown in Figure 8c.

#### 4.1.3. Message Flow during In-Cluster Node Position Adjustment

**Figure 9:**
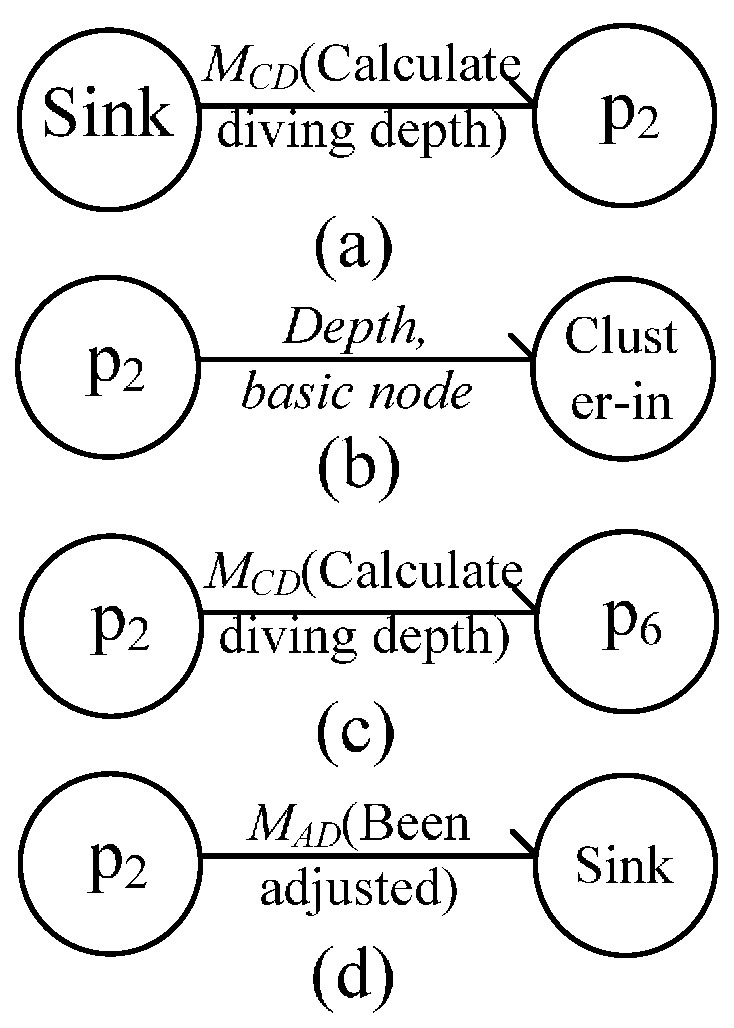
Message flow during position adjustment. (**a**) Initialization phase of position adjustment; (**b**) Phase of position adjustment; (**c**,**d**) End of position adjustment.

In this process, the message flow between nodes takes place in three sub-processes. First, a cluster head node receives *M_CD_* from its next-hop node and begins to calculate the position of its in-cluster nodes, such as *p_2_* in Figure 6c. Second, after the position calculation of in-cluster nodes, it sends message to its in-cluster nodes. Last, it sends *M_CD_* to one of its last-hop nodes if it cannot be adjusted. Otherwise, the node sends *M_AD_* to its next-hop node. The message flows between them are respectively shown in Figure 9a–d.

### 4.2. Complexity Analysis

The message and time complexity of URSA are evaluated in this section.

#### 4.2.1. Message Complexity

The total number of sent messages is considered to determine the message complexity. The message complexity includes three parts, according to the analysis of the message flow between nodes in Section 4.1.

*N* nodes are scattered on the water surface randomly and uniformly. At the beginning of the uneven clustering, *N_p_* nodes become provisional cluster head nodes, and each of them broadcasts its own status message (node ID, *d*(*p_i_,Sink*), *R*(*p_i_*)), where *N_p_* is formulated in Equation (17) according to Equation (8).

(17) $$N_{p}=\frac{N\pi d_{hot}^{2}\left(T_{h1}+3T_{h2}-4T_{h3}\right)}{length\times width}+NT_{h3}$$

Each of them then makes a decision by broadcasting *M_CH_* to act as the final cluster head node or *M_GC_* to exit the competition. Assuming that there are *K_c_* cluster head nodes, they send *K_c_* “elect cluster head” messages, and then (*N* − *K_c_*) non-cluster head nodes send (*N* − *K_c_*) “join” messages. Thus, in the process of the uneven clustering, the total number of message is formulated as:

(18) $$2N_{p}+K_{c}+\left(N-K_{c}\right)=2N_{p}+N$$

During the process of the path selection, each cluster head node sends a request message to its next-hop node, and then, a corresponding reply message is sent to it. Thus, the total number of messages is 2*K_c_* in the process.

In the phrase of in-cluster nodes’ position adjustment, each cluster head node calculates the positions of in-cluster nodes, when it receives *M_CD_* from its next-hop node. After the positions of nodes in its cluster are adjusted, it sends *M_AD_* to its next-hop node sooner or later. Therefore, there must be two messages transmitted on the link between a cluster head node and its next-hop node. What is more, each cluster head node has only one next-hop node because of the hybrid radius path-selection method. Therefore, the network has *K_c_* cluster head nodes, and a sink node has *K_c_* links; and the number of message transmitted on these links is 2*K_c_*. In addition, each cluster head node respectively sends a corresponding adjustment message to its in-cluster nodes, after calculating the depths of nodes in its cluster. Supposing that the *i*-th cluster has *N_i_* in-cluster nodes, the number of messages in the sub-process is formulated as:

(19) $$\sum_{i=1}^{K_{c}}N_{i}=N$$

Finally, the total number of the whole process is 2*K_c_* + *N*.

Consequently, the total number of URSA messages is formulated as:

(20) $$2N_{p}+N+2K_{c}+2K_{c}+N=2N_{p}+2N+4K_{c}<8N$$

Thus, the message complexity of URSA is *O*(*N*).

#### 4.2.2. Time Complexity

At the beginning of the uneven clustering, each node synchronously produces a random number from zero to one and estimates itself to be a provisional cluster head node or not, and then, each of the provisional cluster head nodes synchronously broadcasts its status message. The time complexity of these operations is constant. In the cluster head competition phrase, a provisional cluster head node may become a final cluster head node, which needs to compare the distance to the sink node with all of its competition neighbor nodes or exit the competition. For the convenience of expression on time complexity, the number of competition neighbor nodes of a provisional cluster head node is expressed by the average number of competition neighbor nodes in the network, signed as *N_a_*. Consequently, the time complexity of the operation that a provisional cluster head node becomes a final cluster head node is *N_a_*, and the time complexity of the operation that it exits competition is constant. However, the worst situation in the phrase is that each of them judges itself to be a final cluster head node or not in the one-by-one way, and the time complexity of the situation is *N_a_* × *K_c_* + *N_p_* − *K_c_* + 2. At the end of the uneven clustering, each non-cluster head node joins a final cluster head node that is the nearest to it. The worst situation is that an ordinary node receives *K_c_* messages from all final cluster head nodes, so that it will compare the distance *K_c_* times; consequently, the time complexity is (*N* − *K_c_*) × *K_c_*. Thus, the time complexity of the uneven clustering is formulated as Expression (21), *i.e.*, *O*(*N^2^*).

(21) $$N_{a}\times K_{c}+N_{p}-K_{c}+\left(N-K_{c}\right)K_{c}+2<N^{2}+N_{p}-K_{c}+N^{2}+2$$

During the process of the path selection, the worst situation is that each cluster head node selects ordinary nodes within *R­_c__Max* as its next-hop node. In this situation, the time complexity of the *i*-th cluster head node is *N_i_*. Thus, the time complexity of the total process is max(*N_i_*), *i.e.*, *O*(*N*), because each cluster head node finds its next-hop node synchronously.

In the phrase of in-cluster nodes’ position adjustment, the cluster head node calculates the depth of every in-cluster node. Assuming that the *i*-th cluster has *N_i_­* in-cluster nodes, the worst situation is that an in-cluster node has *N_i_* basic nodes, and for every basic node, the cluster head node should calculate the *CRR* of 2*R_c__Max* positions. Then, it may select the minimum *CRR* from the *CRR* of *N_i_* basic nodes. What is worse, there are *N_i_* basic nodes whose *CRR* is within *rang* range of the minimum *CRR*; in other words, the cluster head node should select the basic node with minimum hops from *N_i_* basic nodes. Thus, the time complexity of the whole process is formulated as:

(22) $$\begin{array}{cc} \sum_{i=1}^{K_{c}}N_{i}\left(2R_{c}\_Max\times N_{i}+N_{i}+N_{i}\right) & =\left(2R_{c}\_Max+2\right)\sum_{i=1}^{K_{c}}N_{i}^{2}\\ & <\left(2R_{c}\_Max+2\right){\left(\sum_{i=1}^{K_{c}}N_{i}\right)}^{2}\\ & =\left(2R_{c}\_Max+2\right)N^{2} \end{array}$$

That is, the result is *O*(*N^2^*).

Finally, the time complexity of URSA is *O*(*N^2^* + *N* + *N^2^*), *i.e.*, *O*(*N^2^*).

## 5. Simulation and Performance Analysis

### 5.1. Simulation Scenario and Parameter Settings

In this study, the four indicators of URSA and CDA, namely, network coverage rate, network energy consumption, network reliability and energy consumption balance, are used to simulate, analyze and compare, in order to verify the validity of URSA. The UWSNs node-deployment process is simulated by MATLAB on the basis of the background of the Xixi Wetland water environment monitoring. During the simulation, the target water area (length × width × depth) is set to 400 m × 200 m × 500 m, and the value of each indicator is produced by the average of 30 rounds of simulation data. The parameter settings are as follows:

(1) The parameter settings are based on the CDA in [26], *i.e.,* the sensing radius of nodes *R_s_* is 40 m, and the communication radius of nodes *R_c_* in CDA is 100 m. This study also considers the situation wherein the distance between two nodes is greater than 2*R_s_*; thus, the maximum communication radius *R_c__Max* in URSA is also set to 100 m.
(2) According to [38], a proportionality constant *c* influences the uneven clustering results, namely if the value of *c* is small, the cluster size is small, and the number of clusters is large. The purpose of the current study is to appropriately increase the number of clusters near the sink node; hence, *c* is set to 0.7.
(3) According to the method of calculating the optimum radius of hotspots *d_hot_* in [39] and the parameter of the simulation scene set in the current study, *d_hot_* is set to 50 m.
(4) Other parameters are presented in Table 1.

**Table 1:** Simulation parameters.

Parameter	Value
Length of data packet *l*	1000 bits
Energy consumption of data reception *P_r_*	2 nJ/bit
Energy diffusion factor *λ*	1.5
Carrier frequency *F_r_*	24 kHz
Data transmission speed underwater	300 bits/s
Initial competition radius *R_0_*	80 m
Probability threshold parameter *T_h1_*, *T_h2_*, *T_h3_*	0.8, 0.6, 0.5
Parameter *k*	3

### 5.2. Simulation Example

#### 5.2.1. Effect of *rang* on Network Reliability and Network Coverage Rate

**Figure 10:**
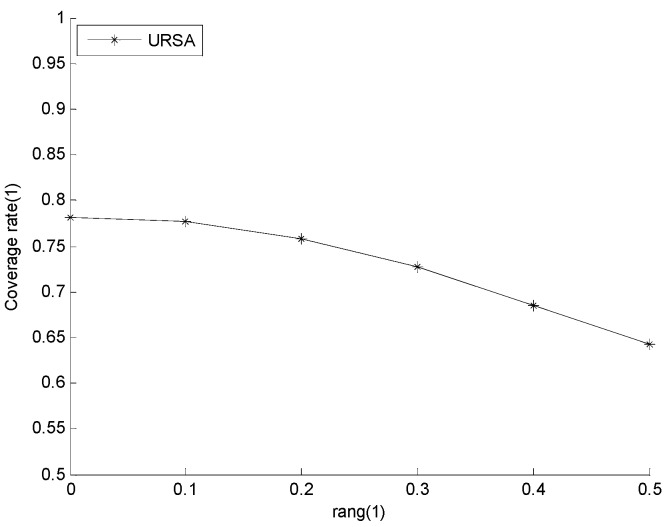
Network coverage rate of the radius-adjusting self-deployment algorithm (URSA) for varying *rang*.

**Figure 11:**
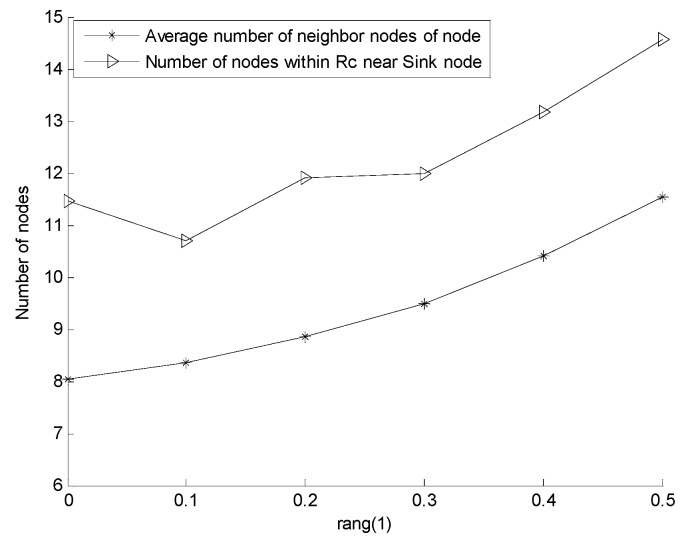
Network reliability of URSA for varying *rang*.

During the adjustment of the depth of in-cluster nodes, the cluster head node finds the optimum location for its in-cluster nodes within the *rang* of the minimum *CRR*; thus, the value of *rang* has an influence on the results of network deployment. This study explores the changes in network coverage rate and network reliability when the number of network nodes is 200 and the value of *rang* varies from zero to 0.5. Figure 10 shows the network coverage rate of URSA for varying *rang*, in which the network coverage rate decreases and the rate of decrease increases with increasing *rang*. Figure 11 presents the network reliability of URSA for varying *rang*, in which the number of nodes within the *R_c_* range of the sink node immediately shows an inconsiderable fluctuation. However, its overall tendency is growing, and the rate of increase increases gradually with increasing *rang*. The average number of neighbor nodes increases all of the time, and the rate of increase accelerates with increasing *rang*. When the cluster head node selects the best locations for its in-cluster nodes, the possibility dominated by the number of hops increases and the possibility dominated by the value of *CRR* decreases with increasing *rang*. This phenomenon increases the number of nodes near the cluster head node, shortens the path in the cluster, increases the chance of the node close to the sink node and increases the possibility of nodes to gather. Therefore, an increasing *rang* improves the network reliability, but reduces the network coverage rate. The preceding analysis implies that the value of *rang* can be set according to the importance of each indicator in the actual application. In this study, considering the compromise between the network coverage rate and network reliability, *rang* is set to 0.2 in every example below.

#### 5.2.2. Effect of Varying *N* on Every Indicator

**Figure 12:**
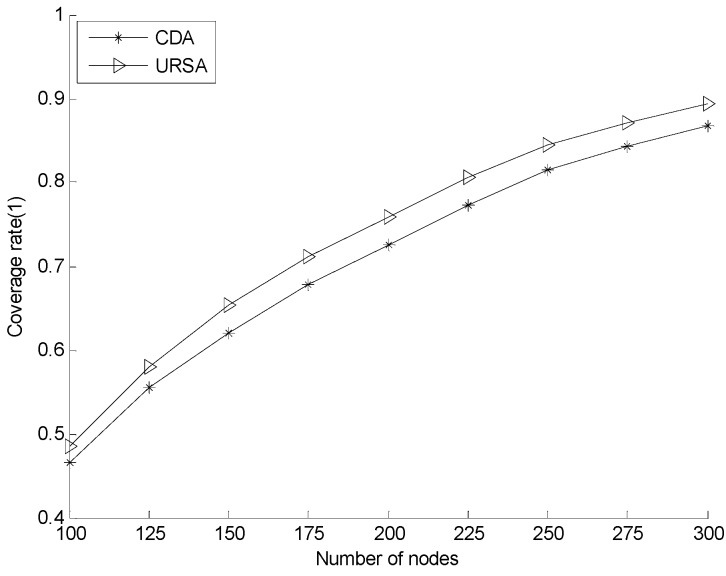
Comparison of network coverage rate for varying numbers of nodes. CDA, CDS-based depth computation algorithm.

Figure 12 presents the comparison of the network coverage rate with varying numbers of nodes. The figure shows that the network coverage rates of both URSA and CDA increase, but the trend gradually slows down with the increasing number of nodes. The network coverage rate of URSA is always higher than that of CDA with the same number of nodes, because CDA only considers the *CRR* on the position where the distance between the node and each of its basic nodes is *R_c_* and does not consider the situation of the other positions where the distance between them is less than *R_c_*. In the situation that CDA does not consider, a node may overlap in such a position. However, the total *CRR* rate of the node may be less than that in the situation that CDA can consider. In URSA, the process of node adjusting depth considers the aforementioned limitation, compares the *CRR* of all possible positions and selects the best position. Therefore, under the circumstances of losing some network coverage rate because of improving the network reliability, the network coverage rate of URSA remains higher than that of CDA.

**Figure 13:**
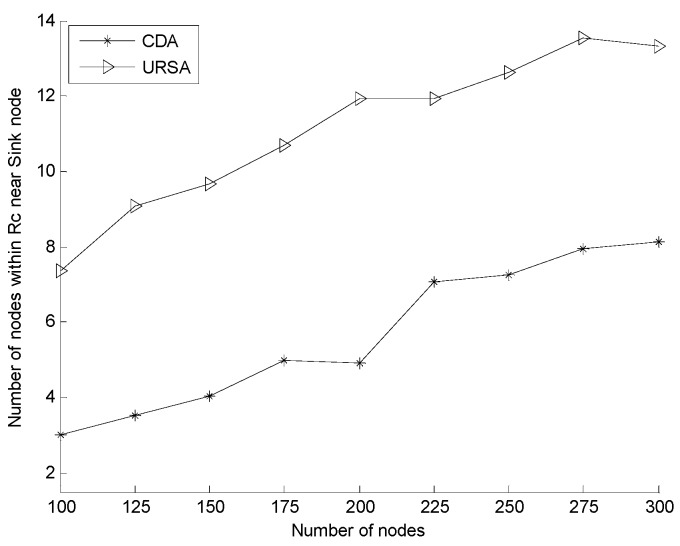
Comparison of the node number within the *R_c_* range of the sink node for a varying number of nodes.

**Figure 14:**
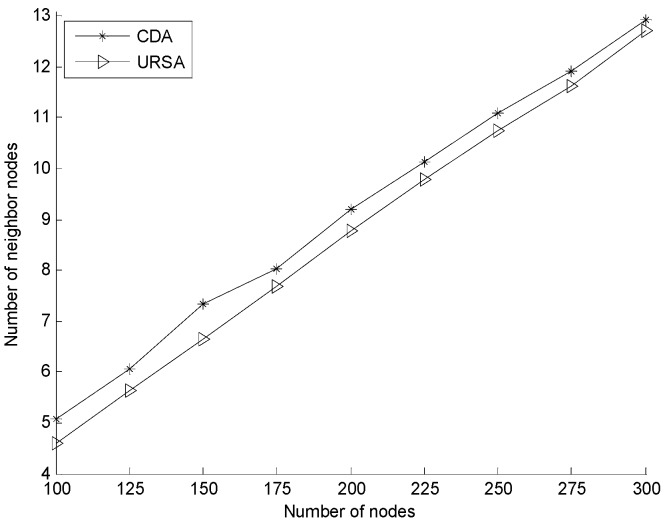
Comparison of the average number of neighbor nodes for a varying number of nodes.

Figure 13 illustrates the comparison of the number of nodes within the *R_c_* range of the sink node for the varying numbers of nodes. In the figure, the number of nodes within the *R_c_* range of the sink node generally increases in both of the two algorithms. However, compared to CDA, URSA shows improved results with the same number of nodes. URSA uses the uneven clustering process to form a layout in which the number of cluster head nodes in an area close to the sink node increases, whereas the size of the cluster in that area decreases. URSA adjusts the position of the cluster head nodes according to the principle of maintaining this layout to increase the number of cluster head nodes near the sink node. The size of the cluster in the area close to the sink node becomes small, and all in-cluster nodes adjust their depth by minimizing their hop number, thereby increasing the chance for in-cluster nodes close to the sink node to some extent. Figure 14 shows the comparison of the average number of neighbor nodes for a varying number of nodes. The figure shows that the average number of neighbor nodes of the two algorithms increases with increasing the number of nodes. The number of URSA is always smaller than the number of CDA, but the difference between URSA and CDA becomes insignificant gradually with the increasing number of nodes. Figure 13 and Figure 14 indicate that compared to CDA, the average number of neighbor nodes of URSA is low. Nevertheless, the number of nodes within the *R_c_* range of the sink node of URSA is obviously higher than that of CDA, thus maintaining the good performance of the network during its operation after being deployed.

The network energy consumption includes the energy consumption of network deployment and the energy consumption of network operation. According to [40], the energy consumption of a moving node is significantly greater than the energy consumption of the communication among nodes during node deployment. Thus, this study only regards the energy consumption generated by nodes adjusting their depth as the energy consumption of network deployment. For the energy consumption of network operation, its value is usually related to the routing algorithm or protocol that the network used when it operates. However, when the number of nodes is relatively sparse, the problem of the energy consumption of the network operation is related to the topology formed by the node self-deployment algorithm, which is the problem that this paper considers. Therefore, in the simulation, the networks deployed by URSA and CDA use the corresponding topology formed by themselves when they operate. We use the energy consumption model in [36] to calculate the average energy consumption of the node of each round. According to [16], the diving speed of nodes is 2.4 m/min, and the power of nodes is 0.6 w.

**Figure 15:**
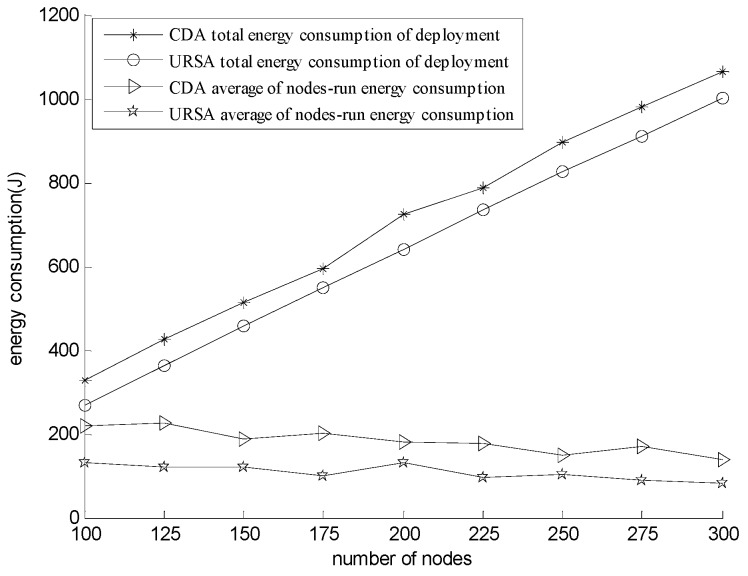
Comparison of energy consumption for varying numbers of sensor nodes (the unit of deployment energy consumption is 10^3^ J).

Figure 15 presents the comparison of network energy consumption for varying numbers of nodes. The figure shows that the energy consumption of the network deployment of both URSA and CDA increases with increasing the number of nodes. Nonetheless, the energy consumption of the network deployment of URSA is always below that of CDA. The energy consumption of network operation of the two algorithms also maintains within a certain value with increasing the number of nodes, but the energy consumption of the network operation of URSA is always below that of CDA. CDA selects the position of every node where the distance between the node and each of its basic nodes is *R_c_*, which makes the path to its cluster head node long. On the contrary, URSA deploys the cluster head nodes near the water surface and adjusts the positions of in-cluster nodes with the principle of minimizing their hop number, which makes the path in the cluster short and reduces the energy consumption of network deployment effectively. In the topology formed by URSA, the communication radius of the node that sends numerous data bags is small (*i.e.,* the distance that those data bags transmit is relatively small), whereas the communication radius of the node that sends a few data bags is great. As a result, the energy loss during data bag transmission decreases, as well as the average node energy consumption of network operations in each round.

**Figure 16:**
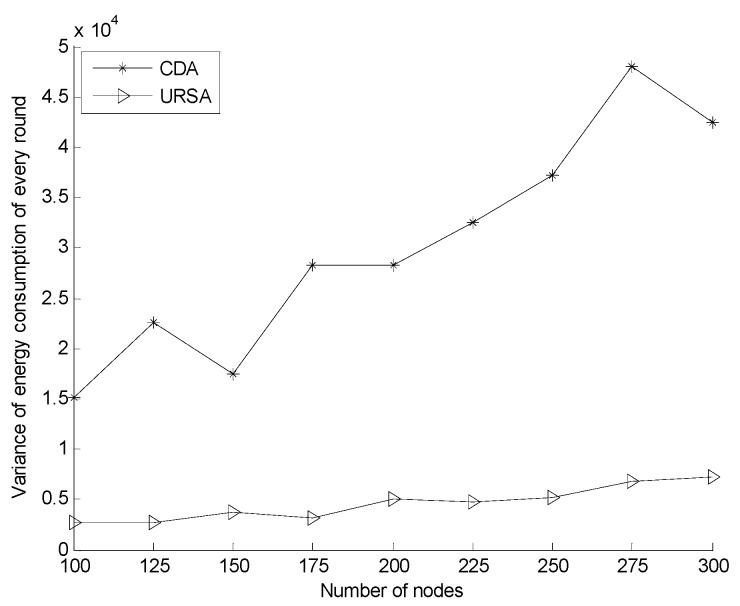
Variance comparison of energy consumption for varying numbers of nodes.

Figure 16 indicates the balance comparison of network energy consumption for varying numbers of nodes. In the figure, the variance of the energy consumption of the network deployed by URSA is significantly below that by CDA owing to the network layout formed by URSA. In such a network layout, the density of nodes in the area close to the sink node is great, whereas the communication radius of the node in that area is small. Nodes, however, should forward considerable information. The situation far away from the sink node is the opposite, which makes the energy consumption of total nodes in the network more balanced. By contrast, using CDA, the communication radius of each node is fixed, and the distribution of nodes in the target area is irregular, thereby leading to the concentrated energy consumption on the nodes near the sink node. With the increasing number of nodes, the amount of information that nodes forward will further increase. Hence, the communication energy consumption of every node cannot be balanced by the different communication radii of nodes, and the variance of the network energy consumption of the two algorithms becomes significant with the increasing number of nodes.

## 6. Conclusions

This study proposes URSA. Each node begins the process of uneven clustering according to the distance to the sink node. Each cluster head node then uses the hybrid radius path-selection method to construct a connected path to the sink node and adjusts its own depth while maintaining the uneven layout on the water surface. For each in-cluster node, a cluster head node considers the redundant coverage rate on the position where the distance between the in-cluster nodes and its basic nodes is greater than 2*R_s_*, equal to 2*R_s_* and less than 2*R_s_* and, finally, selects its position to minimize its hop number on the condition of declining coverage rate. The proposed algorithm, taking into account the indicators of the network coverage rate and network connection rate as the same with the other mobile-restricted self-deployment algorithms, additionally considers network reliability and the balance of the energy consumption of the network during node deployment. In addition, comparing to current algorithms only considering the redundant coverage rate of one or two fixed positions during optimizing the network coverage rate, the proposed algorithm additionally considers the coverage redundancy rate of all positions that the node may reach. The simulation results under different numbers of nodes show that compared to CDA, URSA has a greater network coverage rate, less and balanced network energy consumption and greater network reliability.

As future work, we plan to extend the ideas in this paper considering the node deployment about the node probability perception model and free-to-move nodes. The in-cluster position adjustment strategy needs to be modified to some degree. In addition, we also plan to consider some more realistic models, such as an underwater environment with some obstacles, to improve the algorithm adaptability. Meanwhile, we plan to conduct experiments on the simple sensor nodes and to further verify the algorithm practicability.
